# One-Step Preparation of Nitrogen-Doped Graphene Quantum Dots With Anodic Electrochemiluminescence for Sensitive Detection of Hydrogen Peroxide and Glucose

**DOI:** 10.3389/fchem.2021.688358

**Published:** 2021-06-02

**Authors:** Zheng Yanyan, Jing Lin, Liuhong Xie, Hongliang Tang, Kailong Wang, Jiyang Liu

**Affiliations:** ^1^ Department of Chemistry, Key Laboratory of Surface and Interface Science of Polymer Materials of Zhejiang Province, Zhejiang Sci-Tech University, Hangzhou, China; ^2^ The First Affiliated Hospital of Guangxi University of Chinese Medicine, Nanning, China; ^3^ The First Clinical Faculty of Guangxi University of Chinese Medicine, Nanning, China; ^4^ Affiliated Fangchenggang Hospital, Guangxi University of Chinese Medicine, Fangchenggang, China

**Keywords:** N-doped graphene quantum dots, electrochemiluminescence, anodic, hydrogen peroxide, glucose

## Abstract

Simple and efficient synthesis of graphene quantum dots (GQDs) with anodic electrochemiluminescence (ECL) remains a great challenge. Herein, we present an anodic ECL-sensing platform based on nitrogen-doped GQDs (N-GQDs), which enables sensitive detection of hydrogen peroxide (H_2_O_2_) and glucose. N-GQDs are easily prepared using one-step molecular fusion between carbon precursor and a dopant in an alkaline hydrothermal process. The synthesis is simple, green, and has high production yield. The as-prepared N-GQDs exhibit a single graphene-layered structure, uniform size, and good crystalline. In the presence of H_2_O_2_, N-GQDs possess high anodic ECL activity owing to the functional hydrazide groups. With N-GQDs being ECL probes, sensitive detection of H_2_O_2_ in the range of 0.3–100.0 μM with a limit of detection or LOD of 63 nM is achieved. As the oxidation of glucose catalyzed by glucose oxidase (GOx) produces H_2_O_2_, sensitive detection of glucose is also realized in the range of 0.7–90.0 μM (LOD of 96 nM).

## Introduction

Electrochemiluminescence or electrogenerated chemiluminescence (ECL) is a process in which electrochemical species undergo an electron transfer reaction to form an excited state to emit light (Chen et al., 2011; Li et al., 2017; Zhai et al., 2017; Ma et al., 2020). As an ingenious combination of chemiluminescence and electrochemistry, ECL is currently the most effective analytical technique owing to extraordinary merits of simple instrument and operation, no background signal, high sensitivity, and good controllability (e.g., controlling the reaction by the applied potential at the electrode) (Huang et al., 2021; Ma et al., 2021; Wang et al., 2021).

The ECL emitter (probe) is critical to the analytical performance of the ECL system. In recent years, the development of functional nanomaterials such as ECL emitters has attracted much attention (Yao et al., 2017; Chen et al., 2018). In comparison with the conventional molecular emitters (e.g., metal–organic complexes or organic compounds), nanomaterials, especially quantum dots (QDs), are promising emitters owing to their extraordinary properties and functions, such as a tunable structure and luminescent properties, easy coupling with functional ligands (e.g., protein or DNA aptamer), large specific surface area, and possible catalytic effect (Xu et al., 2014; Bertoncello and Ugo, 2017). However, many QDs are composed of heavy metal elements (such as Cd), which might cause serious health and environmental problems due to inherent biological toxicity of heavy metals. Therefore, a QD-based emitter with excellent luminescence performance and biocompatibility is highly desirable.

Graphene quantum dots (GQDs), as a very promising zero-dimensional (0D) graphene material, are the latest members of the high-value nanocarbon family (Chung et al., 2019; Cui et al., 2020; Qu et al., 2020; Younis et al., 2020; Zhao et al., 2020). Owing to the special structure including the atomic thickness (single or few graphene layers), plane ultrasmall size (less than 10 nm), and sp^2^ carbon structure, GQDs not only exhibit some properties similar to graphene (e.g., high charge transfer, good chemical inertness, and environmental friendliness) but also have unique photoluminescence/electrochemiluminescence properties because of the strong quantum confinement effect (Lu et al., 2019; Yan et al., 2019; Zhang et al., 2019). Compared with organic emitter or semiconductor QDs, GQDs are excellent ECL emitters and have the advantages of a highly adjustable structure and luminescence characteristics, excellent biocompatibility, good stability, and water dispersibility. So far, two different strategies have been developed to prepare GQDs. One is the “top-down” method, that is, cutting large-size graphitized carbon materials through chemical, electrochemical, or physical methods. The other is the “bottom-up” method, which is mainly based on the fusion of small organic molecules under hydrothermal/solvothermal conditions. Compared with the “top-down” strategy, the “bottom-up” method usually has a higher yield and a narrower particle size distribution. In addition, the structure and performance of GQDs can be easily adjusted by changing the precursor, dopant, or modifier in the “bottom-up” method. However, the current method to produce GQDs with the ECL property mainly uses the “top-down” strategy (Zhang et al., 2015; Chen et al., 2016; Zhou et al., 2017; Jie et al., 2019). These methods usually require complex and harsh synthesis procedures. For example, a large amount of acid (e.g., concentrated sulfuric acid or nitric acid) is used for oxidative cutting of graphene, carbon black, or carbon nanotubes into small GQDs. The synthesis also suffers from low production yield. In addition, the as-prepared GQDs mainly have high oxidation states and thus exhibit cathodic ECL at very low potential (‒1.4 V vs. Ag/AgCl) (Lu et al., 2013; Dong et al., 2014; Dong et al., 2015; Zhang et al., 2015). At the same time, there are almost no reports of direct preparation of GQDs with anodic ECL at low potential using the “bottom-up” method. Therefore, efficient synthesis of GQDs with anodic ECL using a simple “bottom-up” strategy remains a great challenge.

**FIGURE 1:**
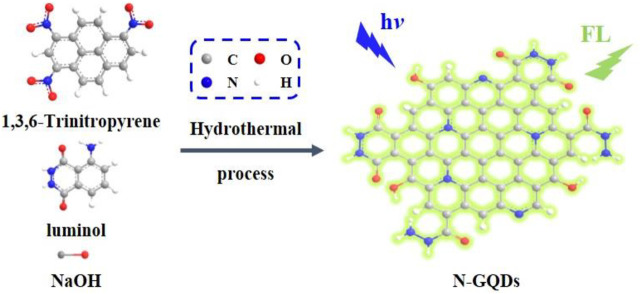
Schematic illustration for one-step preparation of fluorescent N-GQDs using molecular fusion in the hydrothermal process.

In this work, nitrogen-doped graphene quantum dots (N-GQDs) are easily synthesized using one-step molecular fusion in an alkaline hydrothermal process, which enables sensitive sensing based on anodic ECL. As illustrated in Figure 1, 1,3,6-trinitropyrene, a polycyclic aromatic hydrocarbon with a graphene core structure, is applied as a carbon source and luminol as a dopant. The as-prepared N-GQDs have a single-layered graphene structure, uniform size, and high crystallinity. In addition, the applied “bottom-up” method exhibits high production yield. Due to the hydrazide group, N-GQDs exhibit strong anodic ECL properties at low potential in the presence of hydrogen peroxide (H_2_O_2_). With N-GQDs as ECL probes, sensitive ECL detection of H_2_O_2_ is realized. As H_2_O_2_ can be produced by various oxidases and their corresponding substrate, N-GQDs are also employed to detect glucose in combination with glucose oxidase as the proof-of-concept demonstrations.

## Materials and Methods

### One-Step Synthesis of N-GQDs

According to the reports in the literature (Wang et al., 2014), 1,3,6-trinitropyrene was first prepared. For the synthesis of N-GQDs, 1,3,6-trinitropyrene (2 mg/ml) and luminol (0.02 mg/ml) were added to sodium hydroxide (0.1 M). After ultrasonic treatment for 30 min, the resulting ternary mixture was transferred to a polytetrafluoroethylene-lined autoclave and reacted at 180°C for 8 h. After cooling to room temperature, the product was filtered with a microporous membrane (0.22 μM), followed by purification through dialysis with a 500–3,500 dialysis bag. The resulting solution was then freeze-dried to obtain N-GQD power. In order to verify the role of each substance in the synthesis process, 1,3,6-trinitropyrene or luminol was individually treated under the same conditions. The obtained two solutions are defined as control 1 and control 2.

### Characterization

The size of N-GQDs was characterized by transmission electron microscope (TEM, JEM-2100, JEOL Ltd., Japan). The applied voltage was 200 kV, and ultrathin carbon film was used as the supporting film. The thickness of N-GQDs was measured using atomic force microscope (AFM) with a tapping mode (Multi Mode eight type, Bruker. Inc., United States). Freshly peeled mica flake was used as the substrate to deposit N-GQDs. The chemical composition of N-GQDs was characterized using X-ray photoelectron spectroscopy (XPS, PHO5300 type, PE Ltd., United States) at Mg Kα radiation (14 kV) on Au substrate. A fluorescence spectrometer (FL 3C-11, Hariba Scientific, United States) was used to investigate the photoluminescence properties of N-GQDs (Platinum Elmer, Model LS45). The ECL measurements were conducted on an MPI-E II ECL analytical system (Xi’an Remex Analytical Instrument Ltd., China). A traditional three-electrode system was used for electrochemical and ECL measurements. Briefly, glassy carbon electrode (GCE, diameter: 3 mm) was used as the working electrode. Platinum wire acts as the counter electrode, and Ag/AgCl electrode (saturated with KCl solution) was applied as the reference electrode. Before use, GCE was first polished with sandpaper, and then successively polished with 1.0, 0.3, and 0.05 μm alumina slurry.

### ECL Detection of H_2_O_2_ and Glucose

PBS (0.1 M, pH 10.0) containing N-GQDs (50 μg/ml) was applied as the detection medium. For the detection of H_2_O_2_, different concentrations of H_2_O_2_ were added into the detection medium, and ECL signals were recorded. To detect glucose, different concentrations of glucose were first incubated with glucose oxidase at 37°C for 40 min to produce H_2_O_2_. Then, the resulting solution was added into the detection solution, and ECL signals were measured.

## Results and Discussion

### The Strategy for One-Step Synthesis of N-GQDs

The reported GQDs with ECL properties were mainly obtained by cutting large carbon sources, including graphite, graphene oxide, and carbon black. This “top-down” strategy usually relies on the oxidative cutting in harsh conditions, such as high concentration of oxidizing acid or high electrochemical oxidation voltage. However, the synthesis suffers from low production yield, and the as-prepared GQDs have wide size distribution. In addition, the obtained GQDs usually have high oxidation states, leading to cathodic ECL at a very low voltage (such as ‒1.4 V vs. Ag/AgCl). This might limit the application in bioanalysis. In contrast to this, Chen and Chi group synthesized hydrazide-modified GQDs (HM-GQDs) through post-modification of acid cleaved GQDs (Dong et al., 2014; Dong et al., 2015). HM-GQDs were proven to exhibit anodic ECL activities at a low potential (about 0.4 V vs. Ag/AgCl) due to the contained luminol-like units.

Inspired by this research, we try to use luminol as the dopant to establish a one-step synthesis method to synthesize GQDs with anodic ECL performance. As illustrated in Figure 1, 1,3,6-trinitropyrene that consists of four peri-fused benzene rings with a unique carbon skeleton like the primitive cell of graphene is selected as the carbon precursor (Wang et al., 2014). Under alkaline hydrothermal conditions, 1,3,6-trinitropyrene first performs dehydrogenation followed by denitration, leading to molecular fusion and the formation of GQDs. At the same time, denitration can also cause many alkaline species such as ‒NH_2_ and OH^−^ to undergo nucleophilic substitution reactions, resulting in the introduction of functional groups and the doping of heteroatoms. Thus, OH^−^ in the NaOH medium and ‒NH_2_ in the luminol dopant can covalently link on GQDs. On the one hand, GQDs have good water solubility owing to abundant ‒OH groups. On the other hand, the hydrazide group in the luminol unit is beneficial to the anode ECL. Based on the mass of the used carbon precursor, the yield for the synthesis of N-GQDs is 67%. In comparison with the “top-down” method using oxidative cleavage, our bottom-up synthesis method has mild synthesis conditions, easy operation, and high yield, suggesting great potential for the scalable production.

### Characterization of N-GQDs

**FIGURE 2:**
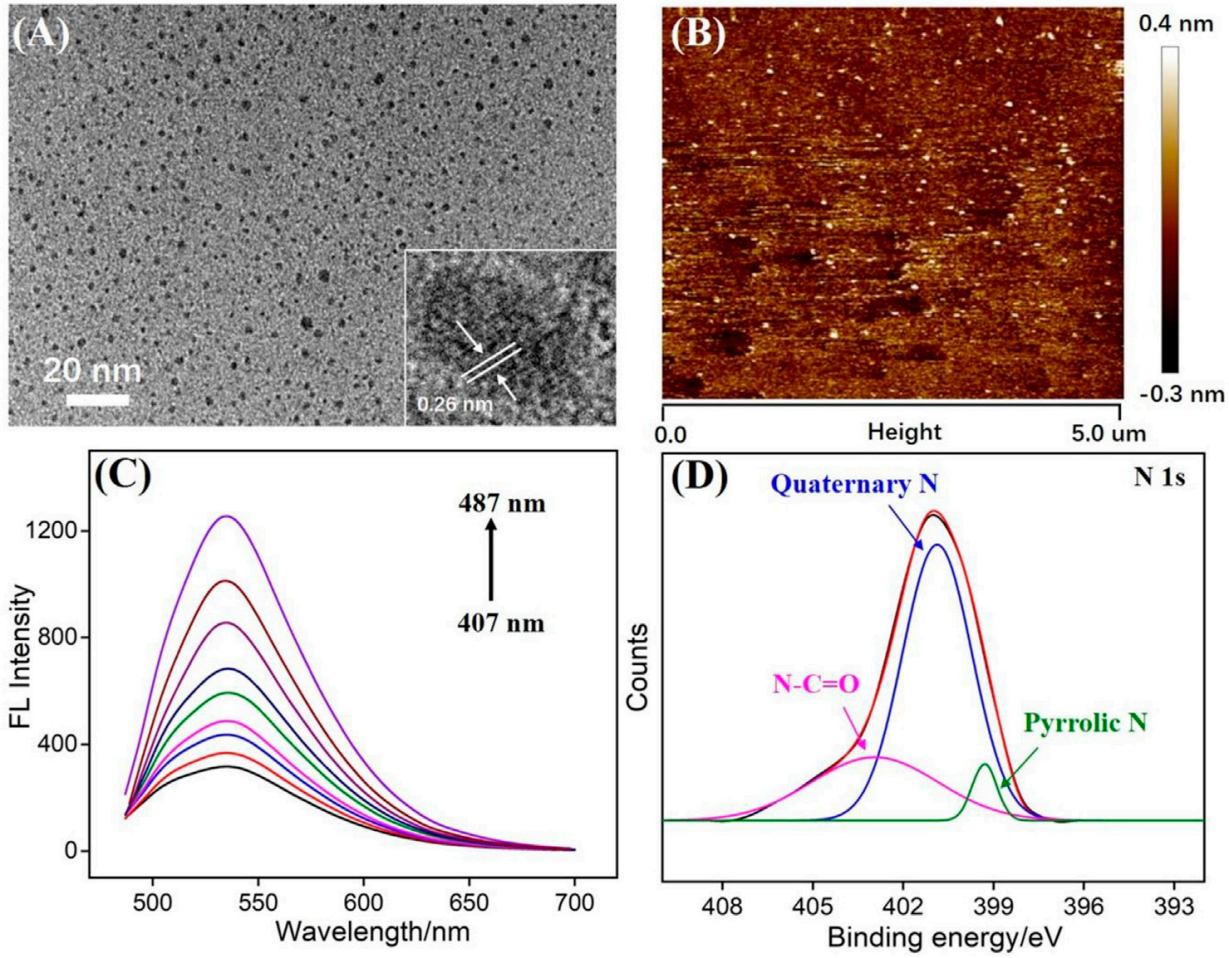
**(A)** TEM images of N-GQDs. Insets present high-resolution TEM (HRTEM) image with resolved lattice. **(B)** AFM image of N-GQDs. **(C)** Fluorescence spectra of N-GQDs obtained with the excitation wavelength ranging from 407 to 487 nm (10 nm increment). **(D)** High-resolution XPS of N1s peaks from N-GQDs.

Transmission electron microscopy (TEM) is applied to characterize the size of N-GQDs. As revealed in Figure 2A, N-GQDs have a relatively uniform size with an average size of about 2.3 nm. In the high-resolution TEM image (HRTEM, inset in Figure 2A), a clear crystal lattice for graphitic carbon is observed, indicating (100) facet of graphene. The thickness of GQDs is investigated using atomic force microscopy (AFM). As shown in Figure 2B, the thickness of N-GQDs is about 0.8 nm (±0.12 nm, 120 samples), indicating the single-layered graphene structure. Different from the zero bandgap of graphene, the energy band of 0D GQDs opens up owing to the significant quantum confinement effect. Thus, GQDs have unique fluorescence properties. Figure 2C is the fluorescence emission spectra of N-GQDs under different excitation wavelengths. Although the excitation wavelength has changed, the maximum fluorescence emission wavelength of N-GQDs (487 nm) has not changed. This proves that N-GQDs have uniform size and surface state.

X-ray photoelectron spectroscopy (XPS) is used to study the chemical composition of N-GQDs. Three elements of C, O, and N can be observed on the XPS spectrum, corresponding to 75.1, 23.3, and 1.6% atomic percentages, respectively (Supplementary Figure S1A in SI). This proves that N-GQDs are rich in oxygen-containing groups and N atoms. The high-resolution C1s spectrum shows that N-GQDs contain sp^2^ carbon (C-C=C), C-O, and N-C=O groups (Supplementary Figure S1B in SI). The high-resolution O1s spectrum indicates peaks corresponding to C=O and C-O/OH groups (Supplementary Figure S1C in SI). In the high-resolution N1s spectrum, N-GQDs are found to contain amide (N-C=O), quaternary N (graphite N), and pyrrole N. Thus, the N doping in GQDs and the presence of functional hydrazide groups are proven. The groups on N-GQDs were also confirmed by Fourier transform infrared (FT-IR) spectrum (Supplemantary Figure S3 in SI). Stretching vibrations of C-O (1,050 cm^−1^), C=O (1,625 cm^−1^), C-N (1,400 cm^−1^), and O-H (3,434 cm^−1^) indicate hydroxyl and amide groups on N-GQDs.

### ECL Behaviors of N-GQDs in the Presence of H_2_O_2_

**FIGURE 3:**
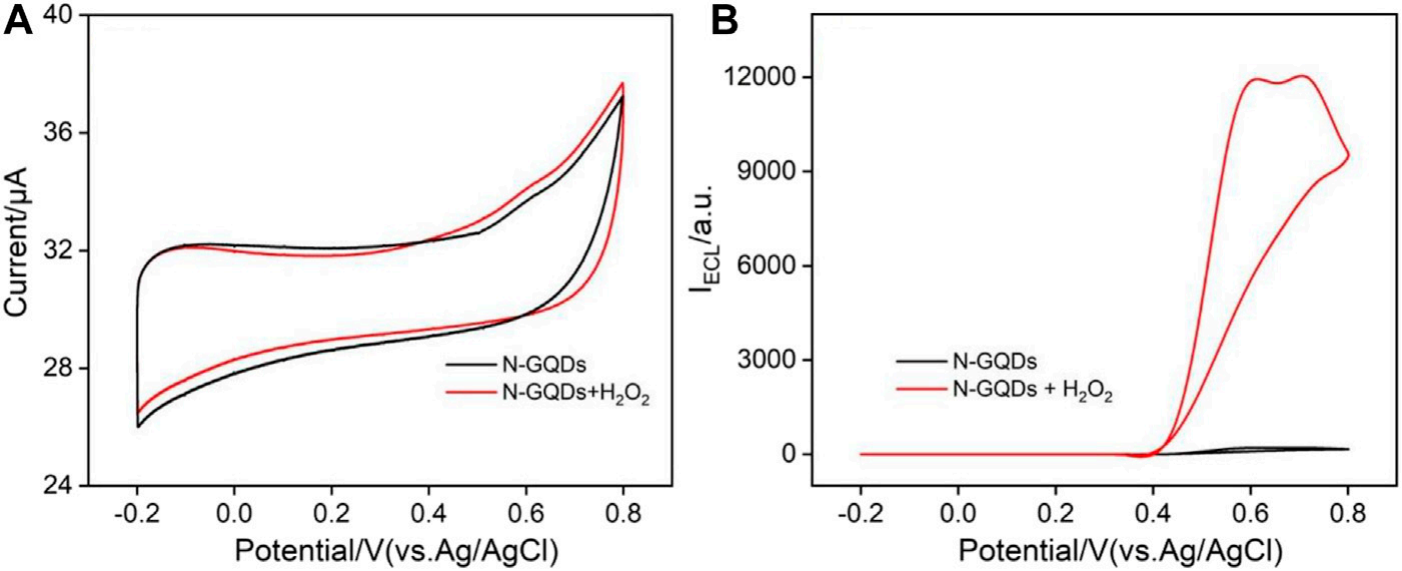
Cyclic voltammograms **(A)** and ECL intensity–potential curves **(B)** of N-GQDs (50 μg/ml) in the absence or presence of H_2_O_2_ in PBS (0.1 M, pH = 10.0).

Figure 3 shows cyclic voltammogram (CV) and ECL–voltage curves of N-GQDs (50 μg ml^−1^) in the absence or presence of H_2_O_2_ in the alkaline PBS medium (pH = 10.0). In comparison with N-GQDs alone, the electrooxidation current of N-GQDs and the H_2_O_2_ system is higher (Figure 3A). Correspondingly, N-GQDs themselves have only weak ECL signals. When hydrogen peroxide is added to the system, the ECL signal is significantly enhanced (Figure 3B). In other words, the addition of H_2_O_2_ increases the ECL signal of N-GQDs by 40 times.

In the present work, 1,3,6-trinitropyrene and luminol are hydrothermally reacted in the NaOH medium to synthesize N-GQDs. Wang et al. reported that hydrothermal treatment of 1,3,6-trinitropyrene alone in the NaOH medium can obtain hydroxyl-modified GQDs (OH-GQDs, control 1). Thus, the luminescent properties of the obtained OH-GQDs are investigated. As revealed in Supplementary Figure S2, OH-GQDs have no ECL signal in the presence of H_2_O_2_, although they have a fluorescence signal like N-GQDs. At the same time, the solution obtained by the same alkaline hydrothermal treatment of luminol was also investigated as another control sample (control 2). Although a weak ECL signal is observed, the fluorescent spectrum of the solution (blue fluorescence) is significantly different from that of N-GQDs (green fluorescence), indicating a completely different structure. This indicates that when luminol hydrothermally reacts alone, it may form materials such as carbon dots or GQDs and thus have a weak ECL signal. However, when the mixed solution of 1,3,6-trinitropyrene and luminol is subjected to hydrothermal treatment, luminol is more likely to be used as a dopant to fuse with 1,3,6-trinitropyrene to obtain N-GQDs.

### ECL Mechanism

**FIGURE 4:**
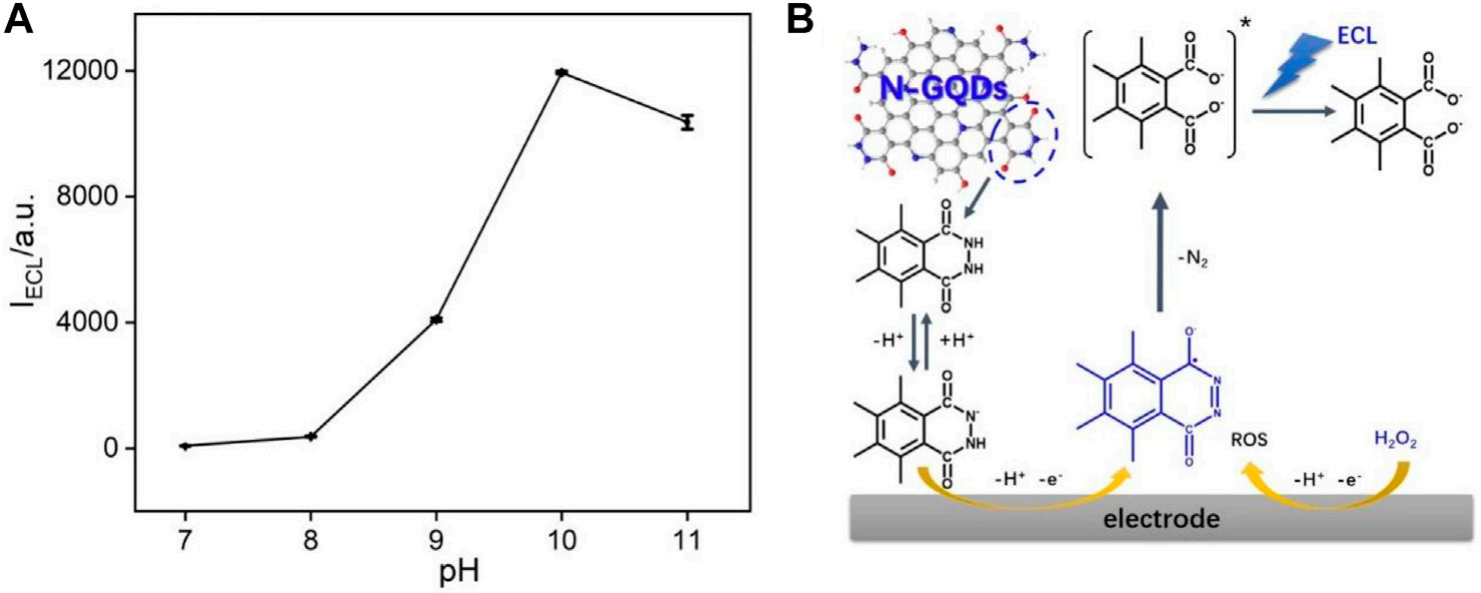
**(A)** Effect of pH on ECL intensity of N-GQDs. **(B)** ECL mechanisms of N-GQDs.

The effect of pH on ECL intensities of N-GQDs in the presence of H_2_O_2_ is investigated. As shown in Figure 4A, the ECL behaviors change significantly with pH. When the pH is lower than eight, the system only has very low ECL signal. The subsequent increase in pH results in a significant increase in ECL intensity. The highest ECL strength is obtained at pH 10. We speculate the possible ECL luminescence mechanism of N-GQDs in the presence of H_2_O_2_. For comparison, the effect of pH on ECL of N-GQDs without H_2_O_2_ has been investigated. As shown in Supplementary Figure S4 in SI, it was clear that pH almost has no effect on the ECL of N-GQDs without H_2_O_2_. Thus, ECL enhancement in the presence of H_2_O_2_ is not coming from the protonation and deprotonation of functional groups on GQDs at different pH values and is due to the presence of H_2_O_2_ (and related reactive oxidative species (ROS)). The cathodic ECL properties of N-GQDs are also investigated. As shown in Supplementary Figure S5 in SI, N-GQDs have negligible anodic ECL. Even in the presence of K_2_S_2_O_8_, the general co-reactant for cathodic ECL of GQDs, only very low ECL intensity is measured. The poor anodic ECL performance might be ascribed to the low oxidative state of N-GQDs resulting from the simple and green synthesis process. As demonstrated in Figure 4B, the abundant luminol-like units in N-GQDs are first oxidized at the electrode to form anion upon anodic potential scanning at GCE. Then, this anion reacts with reactive oxidative species generated by H_2_O_2_ and finally produces the excited-state anion, which emits light (Dong et al., 2014; Dong et al., 2015; Kitte et al., 2017). Thus, electrochemical oxidation of the hydrazide group in N-GQDs and electrogeneration of ROS from H_2_O_2_ are involved in the ECL process. Generally, anodic luminol ECL of N-GQDs is weak in the absence of H_2_O_2_, due to the difficulty of producing ROS. On the other hand, the increase in ECL intensities from pH 8 to 10 is attributed to faster generation of ROS and deprotonation of hydrazide groups. Therefore, PBS at pH 10 is chosen for further investigation to get a sensitive ECL signal.

### ECL Detection of H_2_O_2_

**FIGURE 5:**
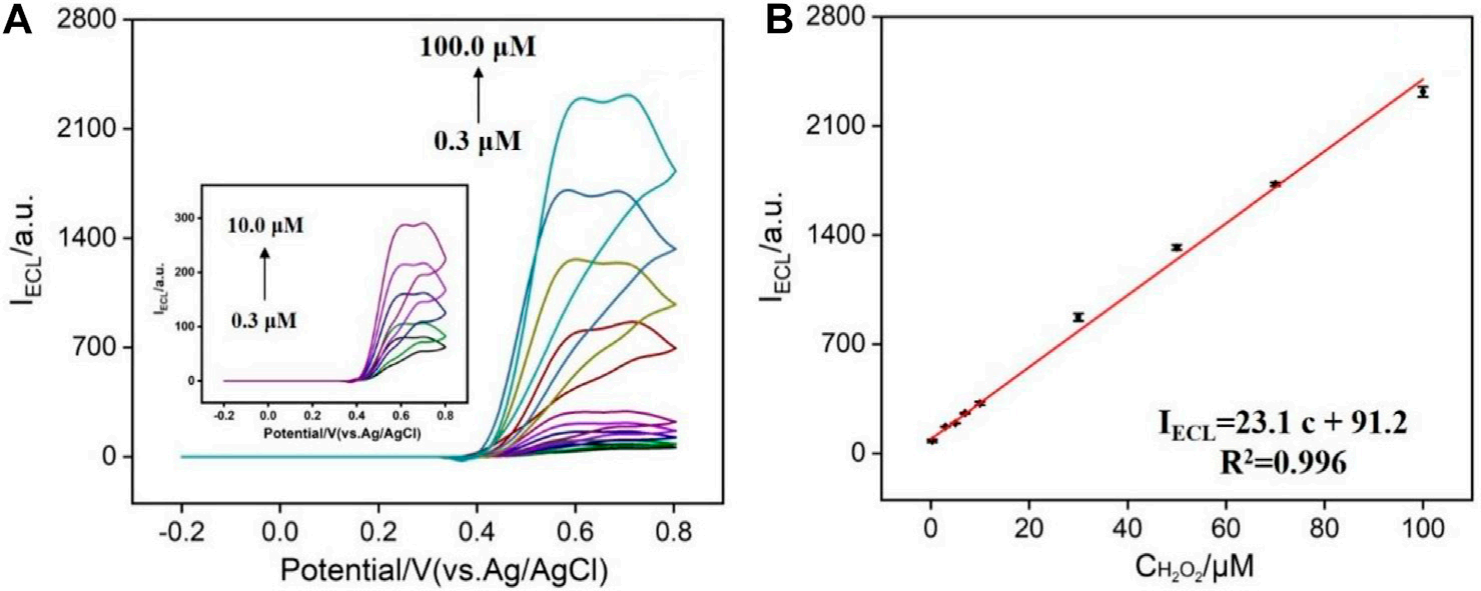
**(A)** ECL intensity of N-GQDs in response to different concentrations of H_2_O_2_ in PBS (0.1 M, pH = 10.0). **(B)** Calibration curve for the detection of H_2_O_2_.

Hydrogen peroxide is an important signal molecule in many biological processes. For example, H_2_O_2_ can be produced by a series of oxides and freely penetrate cell membranes to be widely present in biological tissue compartments (Wang et al., 2015). In addition, hydrogen peroxide is also widely used in textile, leather, paper, wood manufacturing industry, and food manufacturing. Therefore, sensitive detection of H_2_O_2_ is of great significance in bioanalysis, food detection, environmental protection, and other fields. In the presence of H_2_O_2_, the ECL signal of N-GQDs is significantly enhanced, which provides the possibility for the sensitive detection. As shown in Figure 5A, successive additions of different concentrations of H_2_O_2_ lead to the increase of ECL intensity. When the concentration of H_2_O_2_ is in the range of 0.3–100.0 μM, the ECL intensity has a linear relationship with the concentration of H_2_O_2_ (Figure 5B). The limit of detection (LOD) is 63 nM at a signal-to-noise ratio of 3. In comparison with other ECL emitters, N-GQDs have relatively wider detection range and low LOD (Supplementary Table S1 in SI). In comparison with other ECL emitters, N-GQDs have advantages of simple synthesis and good anodic ECL at low potential, suggesting great potential in ECL sensing.

### ECL Detection of Glucose

**FIGURE 6:**
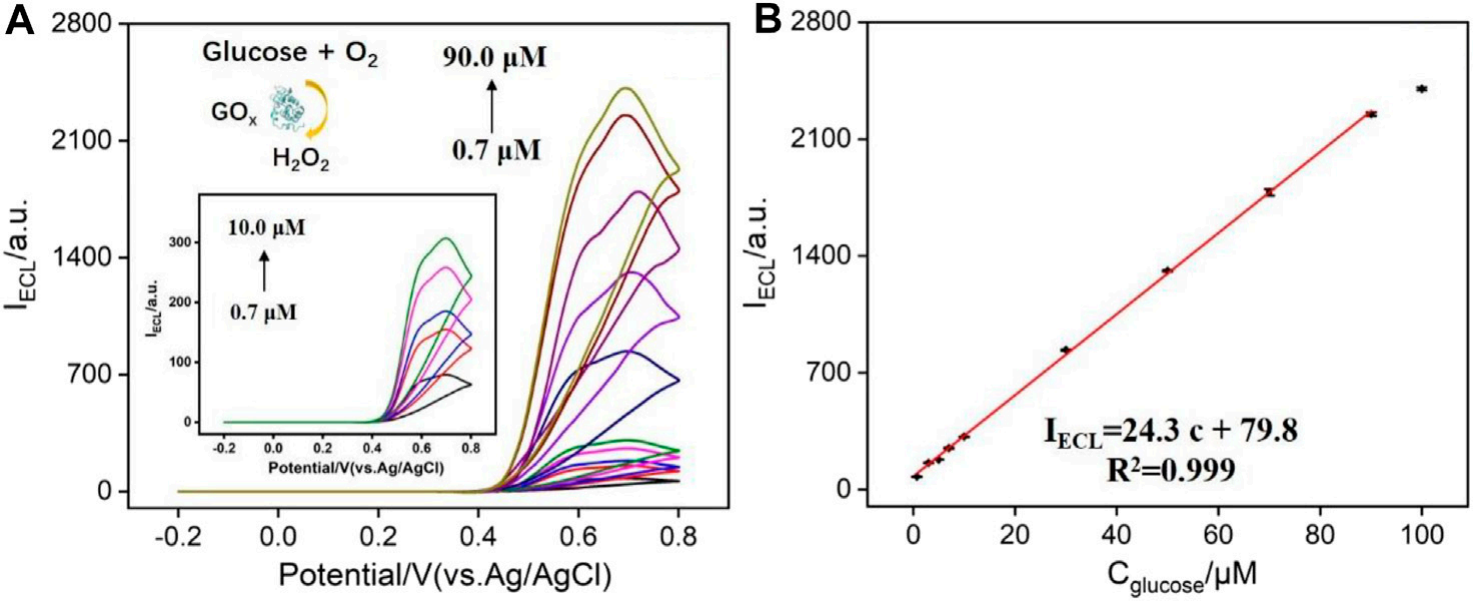
**(A)** ECL intensity of N-GQDs in response to different concentrations of glucose (preincubated with glucose oxidase) in PBS (0.1 M, pH = 10.0). **(B)** Calibration curve for the detection of glucose.

As known, H_2_O_2_ can be produced by various oxidases and their corresponding substrate. Thus, N-GQDs have great potential for the fabrication of the universal platform to detect any substrates (e.g., large variety of metabolites including glucose, cholesterol, lactate, and choline) of oxidoreductases as long as the enzymatic reaction produces H_2_O_2_. Herein, N-GQDs are also employed to detect glucose in combination with glucose oxidase as the proof-of-concept demonstrations. As demonstrated in Figure 6A, the oxidation of glucose catalyzed by glucose oxidase (GOx) produces H_2_O_2_, which can enhance the ECL of N-GQDs. It can be seen that the higher the glucose concentration, the higher the ECL intensity of N-GQDs. The ECL intensity has a linear relationship with the concentration of glucose in the range of 0.7–90.0 μM (Figure 6B). The LOD is 96 nM at a signal-to-noise ratio of 3. A comparison of the detection of glucose using different ECL emitters is given in Supplementary Table S2 (SI). In comparison with other ECL emitters immobilized on the electrode, the detection using N-GQDs is easy and sensitive.

## Conclusions

In summary, we have developed an electrochemiluminescence-sensing platform based on nitrogen-doped graphenes (N-GQDs), which enables sensitive ECL detection of H_2_O_2_ and glucose at low anodic potential. The established one-step synthesis of N-GQDs is simple, green, and has high synthesis yield. As hydrogen peroxide can significantly increase the anodic ECL strength of N-GQDs, N-GQDs are applied for the sensitive detection of H_2_O_2_. In addition, sensitive detection of glucose is also realized because the oxidation of glucose catalyzed by glucose oxidase (GOx) produces H_2_O_2_. The simple synthesis and good ECL performance in the presence of H_2_O_2_ endow N-GQDs with great potential for the fabrication of the universal platform to detect a variety of substrates of oxidoreductases such as metabolites.

## Data Availability

The original contributions presented in the study are included in the article/Supplementary Material; further inquiries can be directed to the corresponding author.
