# Supplementary material for: One-Step Preparation of Nitrogen-Doped Graphene Quantum Dots With Anodic Electrochemiluminescence for Sensitive Detection of Hydrogen Peroxide and Glucose
Source: Front Chem. 2021 Jun 2;9:688358. doi: 10.3389/fchem.2021.688358 (PMC8207508; doi:10.3389/fchem.2021.688358)
Supplement: Supplementary file 1 [file DataSheet1.docx]

**Supporting information**

**Figures**

**
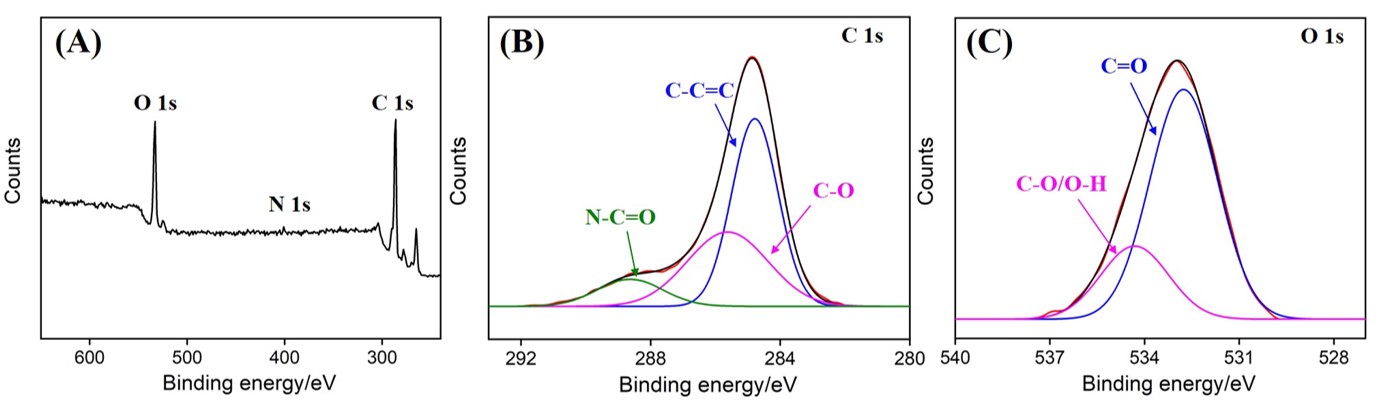
**

**Fig. S1** XPS survey spectrum (A) and high-resolution C 1s (B) or O 1s (C) spectra of N-GQDs.


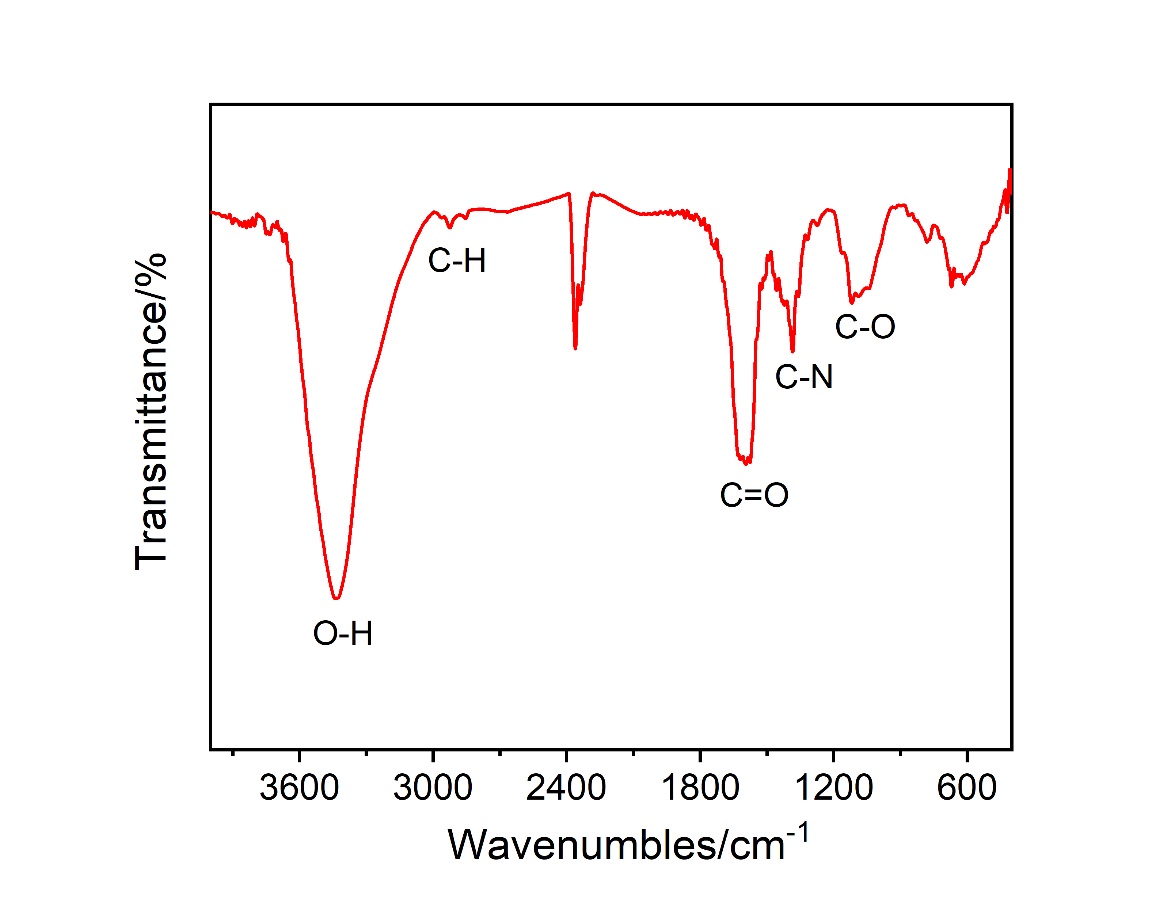


**Fig. S2** FT-IR spectrum of N-GQDs.


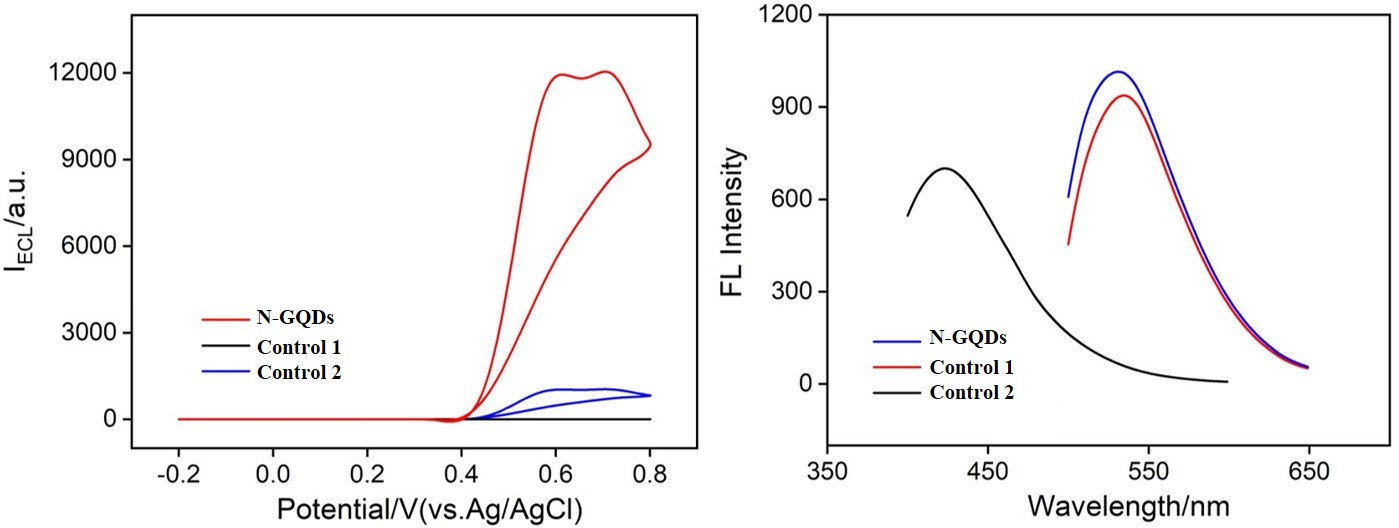


**Fig. S3** ECL intensity-potential curves of N-GQDs and two control samples. 1,3,6-Trinitropyrene or luminol was individually treated under the same conditions for the synthesis of N-GQDs to obtain control 1 and control 2, respectively.


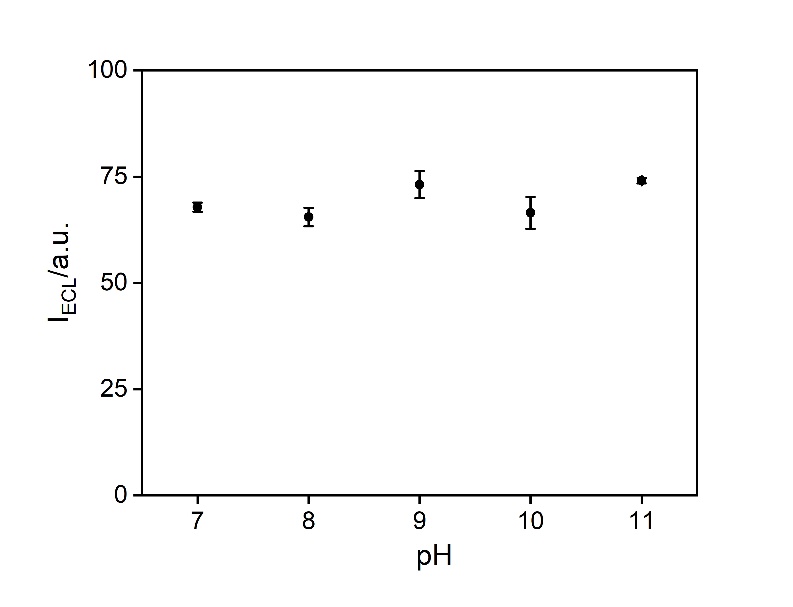


**Fig. S4** ECL of N-GQDs without H_2_O_2_ at different pH values.


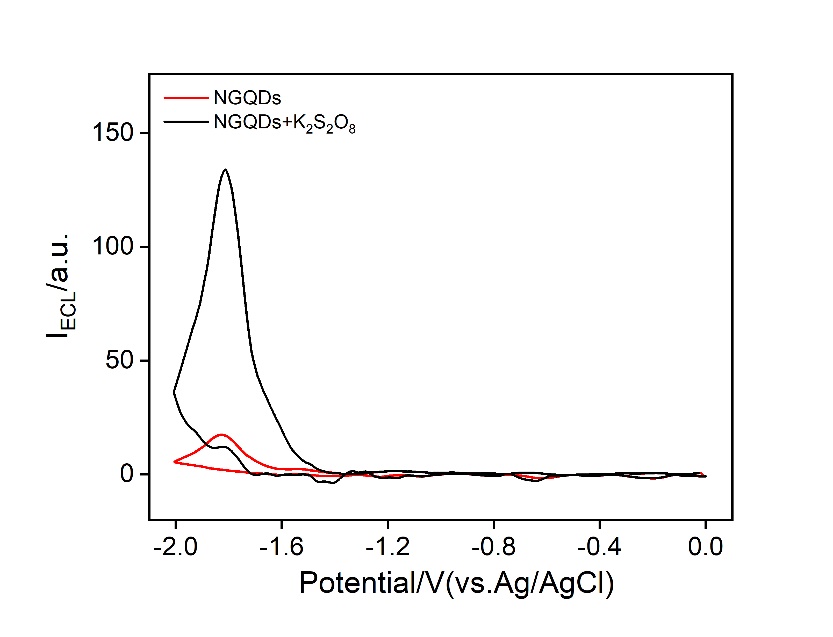


**Fig. S5** ECL intensity-potential curves of N-GQDs in absence or presence of 0.1 M K_2_S_2_O_8_ in PBS (0.1 M, pH=10.0).

**Tables**

**Table S1** Comparison of the detection of H_2_O_2_ using different ECL emitter.

| **System** | **Linear range**  **(μM)** | **Detection limit**  **(nM)** | **References** |
| --- | --- | --- | --- |
| Luminol functional GQDs- | 0.033-74 | 10 | [1] |
| Graphene-CdS nanocomposites | 5-1000 | 1700 | [2] |
| Hydrazide-modified-GQDs | 3-500 | 700 | [3] |
| CdTe QDs | 0.2-10 | 98 | [4] |
| Au/CdS nanocomposite | 0.01-660 | 5 | [5] |
| N-GQDs | 0.3-100 | 63 | this work |

**Table S2** Comparison of the detection of glucose using different ECL emitter.

| **System** | **Linear range**  **(μM)** | **Detection limit**  **(nM)** | **References** |
| --- | --- | --- | --- |
| GQDs | 1.2×10^-6^-1.2×10^-4^ | 3×10^-4^ | [6] |
| CdTe QDs | 0.8-100 | 300 | [7] |
| Au NPs-CdTe QDs | 10-10000 | 5280 | [8] |
| GQDs-AuNPs | 0.1–5000 | 64 | [9] |
| Luminol functional GQDs-AgNP nanocomposites | 25-250 | 8000 | [10] |
| N-GQDs | 0.7-250 | 96 | this work |

References

[1] Tian, K., Li, D., and Tang, T. (2018). A novel electrochemiluminescence resonance energy transfer system of luminol-graphene quantum dot composite and its application in H_2_O_2_ detection. Talanta. 185, 446-452.

[2] Wang, K., Liu, Q., and Wu, X. Y. (2010). Graphene enhanced electrochemiluminescence of CdS nanocrystal for H_2_O_2_ sensing. Talanta. 82, 372-376.

[3] Dong, Y., Dai, R., and Dong, T. (2014). Photoluminescence, chemiluminescence and anodic electrochemiluminescence of hydrazide-modified graphene quantum dots. Nanoscale. 6, 11240-11245.

[4] Wang, Z., Song, H., and Zhao, H. (2013). Graphene-amplified electrogenerated chemiluminescence of CdTe quantum dots for H_2_O_2_ sensing. Luminescence. 28, 259-264.

[5] Shi, C., Shan, Y., and Xu, J. (2010). Enhanced solid-state electrogenerated chemiluminescence of Au/CdS nanocomposite and its sensing to H_2_O_2_. Electrochim. Acta. 55, 8268-8272.

[6] Tian, K., Nie, F., and Luo, K. (2017). A sensitive electrochemiluminescence glucose biosensor based on graphene quantum dot prepared from graphene oxide sheets and hydrogen peroxide. J. Electroanal. Chem. 801, 162-170.

[7] Cheng, L., Deng, S., and Lei, J. (2012). Disposable electrochemiluminescent biosensor using bidentate-chelated CdTe quantum dots as emitters for sensitive detection of glucose. Analyst. 137, 140-144.

[8] Liu, L., Ma, Q., and Li, Y. (2015). A novel signal-off electrochemiluminescence biosensor for the determination of glucose based on double nanoparticles. Biosens. Bioelectron. 63, 519-524.

[9] Wang, D., Liang, Y., and Su, Y. (2019). Sensitivity enhancement of cloth-based closed bipolar electrochemiluminescence glucose sensor via electrode decoration with chitosan/multi-walled carbon nanotubes/graphene quantum dots-gold nanoparticles. Biosens. Bioelectron. 130, 55-64.

[10] Salehnia, F., Hosseini, M., and Ganjali, M. R. (2018). Enhanced electrochemiluminescence of luminol by an in situ silver nanoparticle-decorated graphene dot for glucose analysis. Anal. Methods 10, 508-514.
